# The Feasibility and Validity of Home Spirometry for People with Cystic Fibrosis: Is It Comparable to Spirometry in the Clinic?

**DOI:** 10.3390/children12030277

**Published:** 2025-02-25

**Authors:** Athina Sopiadou, Maria Gioulvanidou, Christos Kogias, Elissavet-Anna Chrysochoou, Ioustini Kalaitzopoulou, Elpis Hatziagorou

**Affiliations:** Pediatric Pulmonology and CF Unit, Hippokration Hospital of Thessaloniki, Aristotle University of Thessaloniki, 54124 Thessaloniki, Greece; athina_sopiadou@hotmail.com (A.S.); maria.gioulvanidou96@gmail.com (M.G.); chrkog4@gmail.com (C.K.); chelianna@gmail.com (E.-A.C.); ioustinek@gmail.com (I.K.)

**Keywords:** telespirometry, cystic fibrosis, spirometry, telemedicine

## Abstract

**Background/Objectives**: Home spirometry allows people with cystic fibrosis (CF) to monitor their lung function from home. However, there are concerns about its feasibility and validity compared to traditional clinic spirometry. The aim of this study was to evaluate the feasibility and validity of telehealth spirometry for patients with CF living in a regional setting. **Methods**: This retrospective study included forty-eight people with cystic fibrosis (pwCF) aged 6–33 years. Participants performed home spirometry using a portable flow sensor spirometer over a one-year period, without supervision. Spirometry readings from portable spirometers were compared with the nearest in-clinic spirometry using the intra-correlation coefficient (ICC) and Bland–Altman plots. Data were collected over a period of one year, with regular intervals of measurements. **Results**: In 427 of the 877 (48.6%) attempted sessions, successful spirometry at home was recorded. Although we showed good reliability between at-home and in-clinic measurements using the Bland–Altman plots and intraclass correlation co-efficient (ICC) (values ranged from 0.76 to 0.88), analysis of the 117 pairs of at-home and in-clinic spirometries showed that mean differences of forced expiratory volume in the 1st sec (FEV1) and forced vital capacity (FVC) obtained at home (both in liter and z-score) had, on average, lower values than the corresponding values at the clinic. **Conclusions**: Home-based telehealth spirometry is feasible among pwCF and provides advantages, especially for those from remote or secluded areas. However, lower values in FVC and FEV1 obtained through home spirometry should not be used interchangeably with clinic values.

## 1. Introduction

Cystic fibrosis (CF) is a severe chronic respiratory disease that requires close monitoring of patients to assess their respiratory function [1]. The most common and accessible way to evaluate the lung function of pwCF is spirometry [2]. During the COVID-19 pandemic, the standard of care protocol for pwCF was disrupted, and many CF centers adopted telemedicine to monitor patients [3].

Telemedicine in CF was introduced by Finkelstein et al. in 1986, with the introduction of a program for monitoring patients at a distance by having a daily journal of symptoms to achieve better survival [4]. Since then, telemedicine has evolved its methods and achieved greater adoption through smartphone software, especially during the COVID-19 quarantine period [5].

Telespirometry is a telemedicine tool used to remotely monitor lung function in patients with chronic respiratory disease. The procedure can be conducted under the supervision of a technician or not, and the results can be transmitted synchronously or asynchronously to the examiner [6]. Despite the promise of home spirometry, questions remain regarding its feasibility and validity compared to traditional clinic spirometry. Are the measurements obtained from home spirometry comparable to those obtained in a clinical setting? Can home spirometry be a reliable tool for monitoring lung function and guiding treatment decisions in CF patients?

Most of the published studies in telespirometry concerning adults with asthma and COPD examine the validity of the measurements taken by portable spirometers and their agreement with the spirometry measurements obtained in a clinic or the office [7,8]. Many CF centers have shared their experience of home spirometry, mainly conducting feasibility studies for adults or teenagers, with only a few of them studying spirometry at home without supervision, as in real-life conditions [3,9,10].

This article explores the feasibility and validity of home spirometry for individuals with cystic fibrosis and examines whether it can be used interchangeably with clinic spirometry.

## 2. Methods

### 2.1. Study Design and Participants

This is a retrospective feasibility study exploring home spirometry in cystic fibrosis patients. We evaluated spirometric data from 48 pwCF older than six years of age who attended our CF clinic over one year during the COVID-19 pandemic (from January 2021 to January 2022). Patients aged 6 to 33 years old were asked to perform spirometry at home twice monthly during stable periods, using a hand-held portable spirometer (MIR SpiroBank smart), and send those measurements to the clinic by email. PwCF and their caregivers (in the case of children < 12 years) were educated on using the portable spirometer over clinic visits and Internet sessions. Moreover, patients performed clinic spirometry with the respiratory laboratory spirometer (Vitalograph 2120) on the standard of care hospital visits, quarterly or sooner if they had symptoms of pulmonary exacerbation. To assess the potential benefits of home spirometry for our patients living in a regional setting, we recorded the distance and the time required to travel to the cystic fibrosis center.

The inclusion criteria of the study population were as follows: (1) Diagnosis of cystic fibrosis confirmed with a sweat test and genetic testing; (2) the ability to perform technically acceptable spirometry in the CF clinic; and (3) the families should have access to the Internet.

The reporting of this study conforms to the Strengthening the Reporting of Observational Studies in Epidemiology (STROBE) statement [11].

The study was approved by the ethics committee, and written informed consent was obtained from all patients in the case of adults or their parents for participants under 18 years of age.

### 2.2. Spirometry

Spirometry data obtained from the measurements were FVC, FEV_1_, ppFEV_1_, FEV_1_/FVC, FEF_25-75_, back-extrapolated volume (BEV), the repeatability of FEV_1_ and FVC, and the grade of the home spirometry according to ATS/ERS 2019 standards. We evaluated pre-bronchodilator spirometries. Successful spirometry was defined as grade A and B.

Clinic spirometry was conducted under the supervision of an experienced nurse in the upright position with no nose clip or bronchodilation, according to ATS/ERS criteria [12]. The analyzed parameters were FVC, FEV1, ppFEV1, FVC/FEV_1_, and FEF_25-75_.

Hand-held portable spirometer MIR SpiroBank smart is a flow sensor with a bidirectional turbine spirometer. It is compatible with the renewed criteria for spirometry of the ATS/ERS 2019 and does not require calibration. The range of flow measurements is ±16 L/s with an accuracy of flow of ±5.0%, or 0.20 L/s, and an accuracy of volume of ±2.5%, or 0.05 L. The laboratory spirometer used is a standard electronic spirometer, a Vitalograph 2120, Lab Manager systemV5 2.0 (Viasys Healthcare, Ennis, Ireland).

Comparison of At-Home and In-Clinic Measurements

The at-home and in-clinic comparison measurements were obtained from 45 patients, with a maximum time difference of 30 days. Two patients were excluded because of continuous unsuccessful spirometries at home; three exceeded the 30-day time limit. From paired data from 117 spirometries, we examined the mean differences in FVC, FEV_1_, and FEF_25-75_ as absolute values and their corresponding z-score (according to 2012 Global Lung Initiative equations, GLI 2012, http://www.lungfunction.org (accessed on 1 March 2022)) [13]. The degree of agreement between the two methods of measuring pulmonary function (spirometry at home and in the clinic) was depicted using the intra-correlation coefficient (ICC) and Bland–Altman plots. A second analysis was conducted for paired measurements with a maximum time difference of 15 days to minimize the possible bias of variability between the measurements due to the significant time difference.

### 2.3. Statistical Analysis

Descriptive statistics were calculated using the mean and standard deviation for continuous variables and frequencies (N) and percentages (%) for categorical variables. No formal sample size calculations were performed; this is a feasibility study. The intra-class correlation coefficient (ICC) and 95% CI for each spirometry parameter were calculated to assess the agreement between measurements taken at home and the clinic. An ICC value above 0.75 was considered good, and a value above 0.90 was considered excellent. Moreover, the Bland–Altman method was used along with the 95% limits of agreement [14]. Limits of agreement quantify dispersion among paired differences, and we defined a range smaller than 150 mL as the cut-off for the clinical acceptance of spirometry according to the ATS/ERS criterion of acceptable repeatability [15,16].

Generalized estimating equations (GEEs) were used to evaluate the association between spirometry evaluation and the different patients’ characteristics: age (6–11 y.o, 12–17 y.o, or >18 y.o), gender (male or female), pseudomonas (none, intermittent, or chronic), disease severity (mild: ppFEV1 > 80%, moderate: ppFEV1 60–80%, or severe: ppFEV1 < 60%) and mutations (homozygous ΔF508, heterozygous ΔF508, or none). The analysis was conducted with IBM SPSS Statistics 28 and a level of statistical significance of 5%.

## 3. Results

Over the study period, 48 pwCF of 6 to 33 years of age, with a mean ppFEV_1_ of 84.98 (StDev 20.94) and a mean distance from the CF Center of 103.99 km (saving 1.5 h per clinic visit), performed 877 home spirometries. The baseline characteristics of the patients are shown in Table 1. According to the ATS/ERS 2019 criteria for acceptability and repeatability, successful assessments were recorded in 48.68% (427/877) of them (Table S1). Generalized estimating equation (GEE) analysis of the association between successful spirometry and the different patients’ characteristics showed that only age was statistically significant.

In the comparative analysis of 117 paired measurements (home vs. clinic spirometries), it was found that FVC and FEV_1_, both in absolute and z-score values, from spirometries performed at home showed lower values on average compared to the corresponding values at the clinic, with a mean difference of −0.37 L (LoA −1.66, +0.96) and −0.22 L (LoA −1.04, +0.60) for FVC and FEV_1_. The mean differences of FEV1 and FVC in z-score were −0.58 (LoA −2.83, +1.59) and −0.76 (LoA −3.89, +2.27), with the z-score being lower for home assessments. For the z-score of the parameters FEF_25-75_ (L/s) and FEF_25-75_, lower values were observed but were not statically significant, as shown in Table 2.

**Figure 1:**
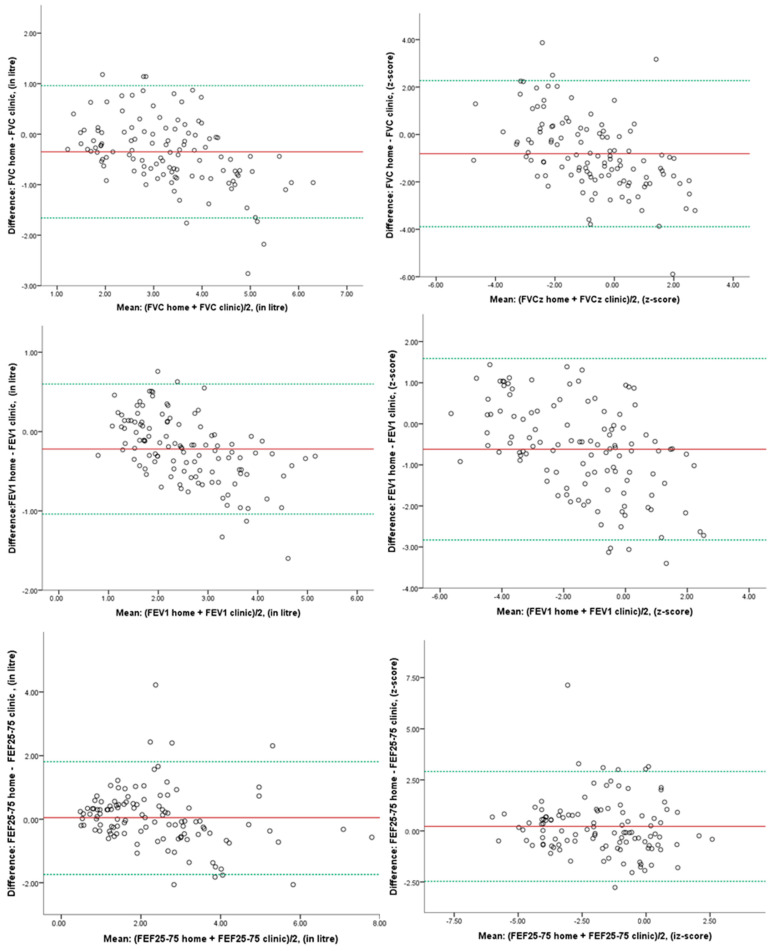
BlandAltman plot of differences in FVC (lt and z-score), FEV_1_ (lt/s and z-score), and FEF_25-75_ (lt/s and z-score) between at-home and in-clinic measurements (red line represents the mean difference and green lines represent the 95% limits of agreement).

Intraclass correlation coefficient (ICC) values ranged from 0.76 to 0.88, indicating good reliability and suggesting that the spirometry parameters were measured similarly at home and the clinic (Table S2). However, for the parameter FVC z-score, the ICC value was 0.57, indicating moderate reliability. In the Bland–Altman plots for each one of the spirometry parameters, there is a small amount of negative bias (Figure 1). However, most differences lie between the limits of agreement, indicating good agreement between spirometry values taken at home and those taken in the clinic.

When a second comparative analysis was conducted for 82 paired measurements from 36 patients with a narrower period between the assessments (maximum 15 days); the results were similar for mean differences, ICC, and limits of agreement (Figure S1).

## 4. Discussion

The study’s primary objective was to examine the feasibility and validity of home spirometry among pwCF by analyzing unsupervised home spirometries performed by 48 patients. We proved that there was good agreement between at-home and in-clinic measurements, using ICC and Bland–Altman plots, independently of the time interval between spirometries. Despite this, FEV1 and FVC differ significantly between the two spirometers, with wide limits of agreement, making their interchangeable use unacceptable.

Of the 877 measurements, 427 (48.68%) were successful spirometries (graded A or B according to the ATS/ERS 2019 revised criteria). It is important to note that despite pwCF being familiar with spirometry and most patients being over 12 years of age, the success rate was still lower than expected. The success rate of home spirometry was lower than expected, which can be attributed to the fact that the spirometry was performed without the supervision of experienced clinic personnel. For example, Kruizinga et al. conducted a prospective study of 90 patients with asthma and CF and found that only about 45% of spirometries conducted at home met ATS/ERS standards [17]. Consistent with our findings, Davis et al. assessed the performance of the Mir Spirobank spirometer in pwCF for unsupervised home spirometry, reporting that 63% of measurements were within acceptable grades (A–C) [18].

Furthermore, the absence of error feedback during the procedure may have contributed to the lower-than-expected at-home spirometry results. Supervised spirometry is crucial for successful spirometry [19]. However, other factors can contribute to lower-quality home spirometry measurements, such as spirometer and diurnal variability differences. Degryse et al., in a validation study of the Mir Spirobank spirometer in a primary care setting, showed that differences in measurements compared to a standard one reach up to 5% [20]. Another comparison study of the MIR spirometer in school-aged children with a history of bronchopulmonary dysplasia depicted lower values in FVC and FEV1 when using the portable spirometer with an acceptable mean bias but with wide limits of agreement [21].

Logie et al. showed that 22 individuals with cystic fibrosis (ages 7–17) achieved a 93% success rate in home spirometry during online appointments with a technician’s guidance [22]. Conversely, Loeb et al. reported a lower success rate of 74% in younger patients (ages 4–17) using a similar protocol [23]. In another study by Alliance involving 937 GPs and 20,757 spirometries, 70% of online assessments met the criteria for excellent and partial cooperation. However, results across telespirometry studies vary, underscoring the importance of supervision during spirometry [24]. Fettes et al. showed that supervised spirometry yielded grade-A results in 89% of measurements compared to 74% in the unsupervised group (*p* < 0.001) [25]. On the contrary, Berlinski et al. found clinically significant differences in FEV1 between coached and uncoached home spirometry compared to clinic measurements, with home spirometry yielding lower values. This highlights the importance of understanding these differences before launching an at-home spirometry program [26].

Comparative analysis of the 117 paired measurements of at-home and in-clinic spirometry proved the good agreement between the two methods for FVC and FEV_1_ with ICC values of 0.80 and 0.88, respectively (*p*-value < 0.001). This relation has been proved in other studies in CF and asthma. The Bland–Altman plots also depicted the good agreement between the two spirometers and showed, as in other studies, the validity of the measurements from home spirometry [22,23,24].

However, the mean difference between FVC and FEV_1_ was −0.37 L and −0.22 L (*p* < 0.001) for paired measurements, indicating the underestimation of the spirometric values in home spirometry and not reaching the ATS/ERS criterion for acceptable repeatability (<150 mL) that most studies define as clinically meaningful [25,27,28]. Paynter et al. studied the mean difference in ppFEV_1_ between a portable spirometer and the spirometer in the clinic in pwCF in cross-sectional and longitudinal aspects. They showed a mean difference of −2.1% in at-home spirometries, expanding to −2.43%, as the period between the two measurements was wider (maximum 28 days) [29]. In accordance with our results, the CLIMB-CF study showed that 76.2% of pwCF recorded lower results in unsupervised home spirometries, with wide limits of agreement in the Bland–Altman plots for FEV1, concluding that at-home and in-clinic spirometry should not be used interchangeably [30]. These results are consistent with those of John Oppenheimer et al. in asthma patients, who proved poor agreement between at-home and in-clinic spirometric measures based on the 95% limits of agreement [31]. In a meta-analysis, Anad et al. demonstrated that, across respiratory conditions, unsupervised home spirometry tends to yield lower values compared to supervised clinic measurements, with wide limits of agreement [32].

Our study also examined the differences in FEV1 and FVC z-score values considering the latest ERS/ATS statement with a mean difference of −0.58 and −0.76, with lower values for at-home spirometry, and clinically significant difference that overrode the 0.5 z-score difference. Unfortunately, few pediatric studies compare portable and standard spirometers and use z-score values [33,34].

### Limitations of the Study

One of the study’s limitations is the population size and the number of spirometries. In our study, we did not employ any reinforcement methods to increase the success rate of the measurements, such as reminders (messages or telephone calls) to increase participation and the total number of assessments at home [35]. The eICE study highlighted that the participation of pwCF in telemedicine studies can be an issue, particularly when frequent measurements (daily or twice per week) are required [36]. To minimize the influence of daily variability typically observed in FEV1, a standard hour should have been defined for conducting home spirometry [37].

## 5. Conclusions

In conclusion, this study examines the feasibility of telespirometry without supervision in an asynchronous way for pwCF. The results suggest that telespirometry can benefit patients living in remote areas, in addition to the standard protocol for monitoring CF patients, by reducing the need for in-clinic visits, lessening travel burden, and lowering infection risk while promoting patient engagement and treatment adherence. In addition, frequent home measurements can be used to track trends over time and to enable early detection of pulmonary decline and timely intervention [37].

Despite the possible benefits of at-home spirometry, the underestimation of FEV1 and FVC values in portable spirometers demonstrates that for now, interchangeable use between spirometers is unacceptable and further research is needed.

## Figures and Tables

**Table 1:** Baseline characteristics of the study population (N = 48).

	N (%), Mean (StDev)
Age	
6–11 y.o	7 (14.58)
12–17 y.o	21 (43.75)
>18 y.o	20 (41.67)
Gender	
Male	22 (45.83)
Female	26 (54.17)
Pseudomonas colonization	
None	35 (72.92)
Intermittent	9 (18.75)
Chronic	4 (8.33)
Disease severity	
Mild (ppFEV_1_ > 80%)	34 (70.83)
Moderate (ppFEV_1_ 60–80%)	8 (16.67)
Severe (ppFEV_1_ < 60%)	6 (12.50)
Mutations	
ΔF508/ΔF508	20 (41.67)
ΔF508/other	20 (41.67)
None	8 (16.67)
Spirometry data	Mean (sd)
FVC (lt)	3.27 (1.03)
FVC pp	94.03 (25.56)
FEV_1_ (lt)	2.59 (0.86)
FEV_1_ pp	84.98 (20.94)
FEF_25-75_ (lt/sec)	2.49 (1.26)
FEF_25-75_ pp	69.89 (29.57)

**Table 2:** Comparison of the spirometry parameters of 43 patients performing at-home and in-clinic spirometry.

	Home Spirometry Mean (SD)	Clinic Spirometry Mean (SD)	Mean Difference (95% CI)	*p*-Value	95% LoA
FVC (lt)	3.27 (1.05)	3.63 (1.35)	−0.37 (−0.54, −0.19)	<0.001 *	−1.66, +0.96
FVC z-score	−0.72 (1.38)	0.03 (1.92)	−0.76 (−1.17, −0.34)	0.001 *	−3.89, +2.27
FEV_1_ (lt)	2.59 (0.91)	2.81 (1.11)	−0.22 (−0.32, −0.12)	<0.001 *	−1.04, +0.60
FEV_1_ z-score	−1.34 (1.58)	−0.77 (2.02)	−0.58 (−0.85, −0.30)	<0.001 *	−2.83, +1.59
FEF_25-75_ (lt/s)	2.50 (1.34)	2.44 (1.40)	0.06 (−0.13, 0.25)	0.526	−1.74, +1.81
FEF_25-75_ z-score	−1.54 (1.67)	−1.72 (1.72)	0.18 (−0.09, 0.46)	0.189	−2.47, +2.91

SD, standard deviation; LoA, limits of agreement; CI: confidence interval. * Statistically significant at the 5% level.

## Data Availability

The data that support the findings of this study are available from the corresponding author upon reasonable request.

## Supplementary Materials

The following supporting information can be downloaded at: https://www.mdpi.com/article/10.3390/children12030277/s1, Table S1: Grading of home spirometries according to the ATS/ERS 2019 criteria for acceptability and repeatability. Table S2: Intraclass correlation coefficient for the reliability between spirometry parameters of home and clinic measurements. Figure S1: Bland–Altman plot of differences in FVC (lt and z-score), FEV_1_ (lt/s and z-score), and FEF_25-75_ (lt/s and z-score) between at-home and in-clinic measurements (the red line represents the mean difference and green lines represent the 95% limits of agreement) in 82 paired measurements from 36 patients with a narrower period between the assessments (maximum 15 days).

## Abbreviations

The following abbreviations are used in this manuscript:

CF	Cystic fibrosis
pwCF	People with cystic fibrosis
ATS/ERS	American Thoracic Society/European Respiratory Society
ICC	Intra-correlation coefficient
FEV_1_	Forced expiratory volume in the first sec
FVC	Forced vital capacity
COPD	Chronic obstructive pulmonary disease
STROBE	Strengthening the Reporting of Observational Studies in Epidemiology
ppFEV_1_	Percent predicted forced expiratory volume in one second
FEF_25-75_	Forced expiratory flow at 25% and 75% of pulmonary volume
BEV	Back extrapolated volume
GLI	Global Lung Initiative equations
GEE	Generalized estimating equations
LoA	Limits of agreement
