# Supplementary material for: The Feasibility and Validity of Home Spirometry for People with Cystic Fibrosis: Is It Comparable to Spirometry in the Clinic?
Source: Children (Basel). 2025 Feb 25;12(3):277. doi: 10.3390/children12030277 (PMC11941756; doi:10.3390/children12030277)
Supplement: Supplementary file 1 [file children-12-00277-s001.zip › children-3472602-supplementary.pdf]

Table S1: Grading of home spirometries according to the ATS/ERS 2019 criteria for acceptability and repeatability

Grading of Home Spirometry	N	% percent
**A**	314	35.80
**B**	113	12.88
**C**	36	4.10
**D**	166	18.92
**E**	2	0.22
**F**	246	28
**Total**	877	100

Table S2: Intraclass correlation coefficient for the reliability between spirometry parameters of home and clinic measurements

	ICC (95% CI)	p-value
**FVC (lt)**	0.80 (0.63, 0.88)	<0.001*
**FVC z score**	0.57 (0.35, 0.72)	<0.001*
**FEV1 (lt/min)**	0.88 (0.76, 0.94)	<0.001*
**FEV1 z score**	0.80 (0.62, 0.88)	<0.001*
**FEF_{25-75} (lt/sec)**	0.81 (0.74, 0.87)	<0.001*
**FEF_{25-75} z score**	0.76 (0.67, 1.83)	<0.001*
ICC: Intraclass correlation coefficient, CI: Confidence interval,<br>*Statistically significant at level 5%		

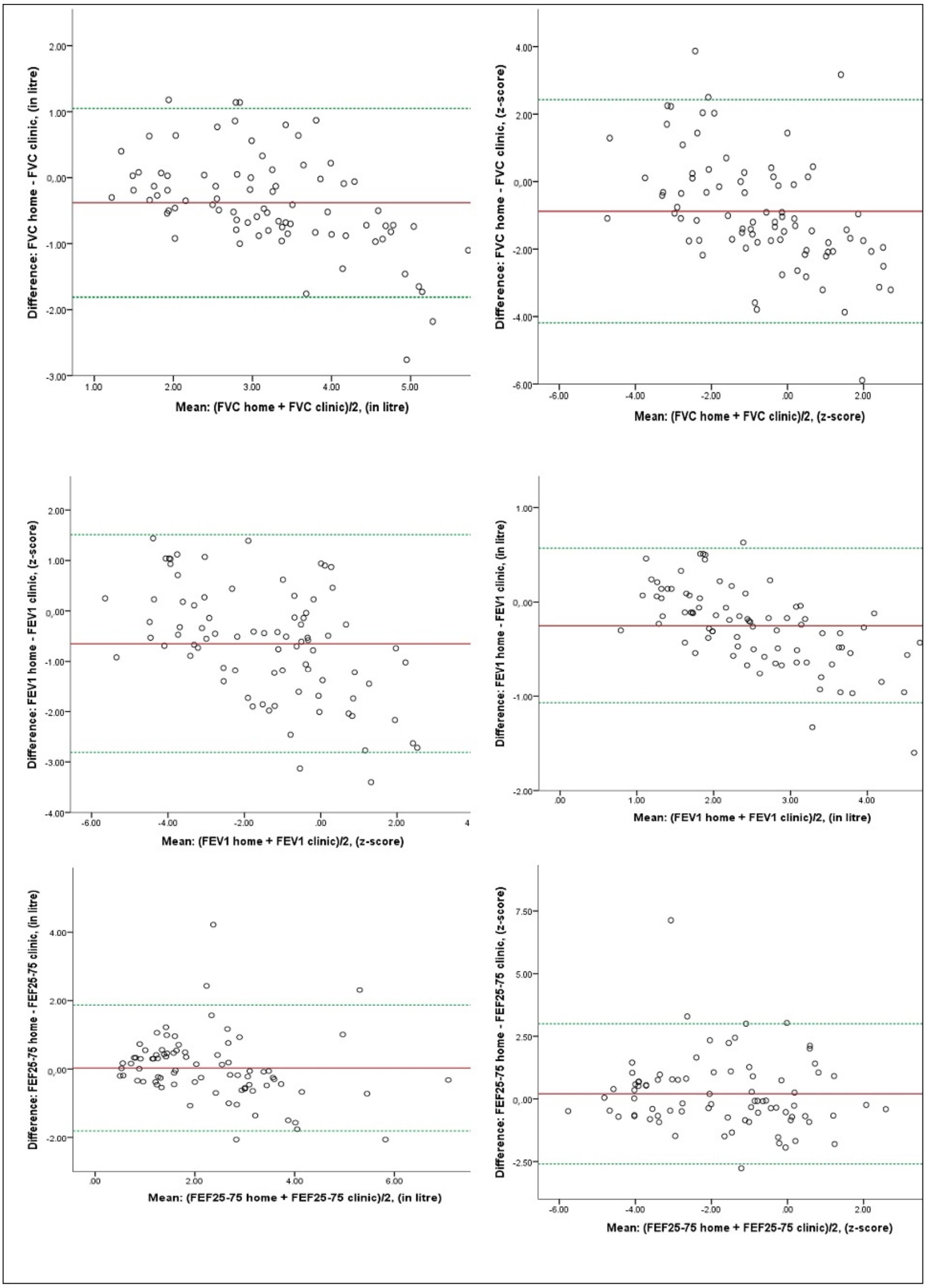

Figure S1. Bland–Altman plot of differences in FVC (lt and z-score), FEV1 (lt/s and z-score), and FEF25-75 (lt/s and z-score) between at-home and in-clinic measurements (the red line represents the mean difference and green lines represent the 95% limits of agreement) in 82 paired measurements from 36 patients with a narrower period between the assessments (maximum 15 days).
